# Screen-Printed Voltammetric Biosensors for the Determination of Copper in Wine

**DOI:** 10.3390/s19214618

**Published:** 2019-10-24

**Authors:** Liliana Norocel, Gheorghe Gutt

**Affiliations:** 1 Department of Health and Human Development, Stefan cel Mare University of Suceava, 13, University Street, 720229 Suceava, Romania; 2 Faculty of Food Engineering, Stefan cel Mare University of Suceava, 13, University Street, 720229 Suceava, Romania; g.gutt@fia.usv.ro

**Keywords:** wine safety, heavy metal, copper casse

## Abstract

Certain heavy metals present in wine, including copper, can form insoluble salts and can induce additional casse, so their determination is important for its quality and stability. In this context, a new biosensor for quantification of copper ions with BSA protein (bovine serum albumin) and using SPE electrodes (screen-printed electrodes) is proposed. The objective of this research was to develop a miniaturized, portable, and low-cost alternative to classical methods. A potentiostat, which displays the response in the form of a cyclic voltammogram, was used in order to carry out this method. Values measured for the performance characteristics of the new biosensor revealed a good sensitivity (21.01 μA mM^−1^cm^−2^), reproducibility (93.8%), and limit of detection (0.173 ppm), suggesting that it has a high degree of application in the analysis proposed by our research. The results obtained for wine samples were compared with the reference method, atomic absorption spectrometer (AAS), and it was indicated that the developed biosensor is efficient and can be used successfully in the analysis of copper in wine. For the 20 samples of red wine analyzed with AAS, the concentration range of copper was between 0.011 and 0.695 mg/L and with the developed biosensor it was between 0.037 and 0.658 mg/L. Similar results were obtained for the 20 samples of white wine, 0.121–0.765 mg/L (AAS) and 0.192–0.789 mg/L (developed biosensor), respectively.

## 1. Introduction

The presence of metallic contaminants in wines is attributed to many endogenous and exogenous factors. The mineral content of wine depends on soil, location, grape varieties, weather or environmental conditions, and wine practices [1]. The main source may be the vineyard sprays and particularly the use of Bordeaux mixture (a copper-based vineyard spray), which significantly contributes to high levels of copper ions in the must (in excess of 20 mg/L in two cases investigated by the Australian Wine Research Institute). However, fermentation usually eliminates much of this excess through binding of the metal to yeast and removal with the lees. Another source would be the contact of a wine with copper or copper alloys during winemaking; furthermore, the addition of copper sulfate to correct sulfidic off-odors is arguably a major cause of copper instability. Certain heavy metals, including copper, can form insoluble salts and can induce additional casse [2]. The presence of copper in high concentration in wine causes the so-called copper casse, which can already be installed at concentrations of 0.5 mg Cu/L, having a negative effect on the organoleptic properties of the wine [1]. Excess of copper ions determined before bottling can be removed from the wine by the addition of potassium ferrocyanide. Therefore, by avoiding wine quality degradation, the determination of these ions has economic benefits for wine producers. Nowadays, most existing methods used for heavy metal trace analysis include spectrophotometric (AAS—atomic absorption spectrometer, ICP/MS—inductively coupled plasma mass spectrometry, ICP/AES—inductively coupled plasma atomic emission spectrometry), voltammetric, and chromatographic methods, which can detect analytes at very low concentrations. However, all these classic techniques are generally costly and laborious, so it is necessary to develop new methods that eliminate these disadvantages. Biosensor systems are relatively recent achievements that allow rapid, inexpensive, and in situ quantitative analytical determinations, providing solutions for food analysis in terms of specificity and time savings [3]. Electrochemical biosensors are often preferred due to simplicity and miniaturization. From this point of view, the use of screen-printed electrodes not only solves the problem of cost efficiency, but also satisfies the portability requirement, contributing to the progress towards decentralized analysis. There are numerous studies on the detection of copper ions with biosensors by the specified means, but most of them are developed around the analysis of these ions in water or foods [4,5,6,7,8]. Only a few studies can be applied in the determination of the copper content of wine, despite the fact that the presence of this element has a great impact on the quality and safety of this product [2,9]. Numerous scientific papers studied the use of bovine serum albumin (BSA) in terms of the mechanism of adsorption and the interactions of the protein with different electrode materials. A series of investigations were particularly focused on the interactions between BSA and copper; these studies are useful for understanding the biological phenomena related to the formation of covalent bonds [10,11,12]. Several investigations used the metal–protein interaction to design electrodes for electrochemical detection of heavy metals [13]. An interesting experiment regarding the binding of Cu (II) ion to BSA was achieved by ion-selective electrode potentiometry, and the results offered more attractive features than some traditional methodologies [14]. The immobilization of proteins on the electrode is important for the development and performance of biosensors and has been investigated in numerous studies [15,16,17,18,19]. The characterization of metal binding to metalloproteins is vital for our understanding of the relationship between structure and function. Fundamental for metal binding activity is the intrinsic binding constant, which is a parameter that describes the affinity with which metal ions are bound to specific protein sites. The central tenet of the strategy is the presentation of the metal to the protein in the form of well-characterized, low-molecular-weight chelates to prevent metal hydrolysis and nonspecific binding. Further, this strategy permits the determination of the number of specific metal binding sites and provides insights into the amino acid ligands involved in binding [20]. Among the immobilization methods applied for the specified recognition element, a simple method is photoreactive immobilization, performed by benzophenone irradiation at the 350 nm wavelength [21,22,23,24]. Benzophenone (Ph_2_CO) is activated by UV light in the range 350–360 nm, and its reaction mechanism is as follows: The carbonyl oxygen forms a ketyl radical when exposed to UV light and then abstracts the hydrogen from a C–H bond in a nearby molecule, followed by C–C bond formation between Ph_2_CO and the attacked molecule. Advantages of Ph_2_CO include chemical stability, activation in a UV range that does not damage proteins or cells, and stability in ambient light [25]. The purpose of this study was to develop a biosensor with practical significance for determining the copper content of wine, using as biological element, BSA protein, immobilized by a photoreactive compound, namely benzophenone, and an electrochemical transducer providing a cyclic voltammogram response. Furthermore, the results obtained by the voltammetric method for 40 wine samples were compared with the reference method, namely AAS (atomic absorption spectrometer), to confirm the efficiency of the developed electrochemical biosensor. The novelty of this biosensor is a new method to determine copper ions in wine which solves the problem of high cost and portability.

## 2. Materials and Methods

### 2.1. Reagents

Standard protein (200 mg BSA/mL), benzophenone (99%), ethanol (98%), ammonium acetate, and copper sulfate were purchased from Sigma-Aldrich (Steinheim, Germany). Screen-printed electrodes (SPE) were purchased from Pine Research (Durham, NC, USA) and include a carbon working electrode with a 2.0 mm diameter, a carbon counter electrode, and a silver/silver chloride (Ag/AgCl) reference electrode. The SPE dimensions are 15 × 61 × 0.36 mm, type RRPE1001C.

### 2.2. Samples

In this study, 40 wine samples (20 samples of red wine and 20 samples of white wine) from different local producers from Vrancea County, Romania were analyzed by voltammetric method. The results obtained were compared with the AAS values of the same samples.

### 2.3. Electrochemical Measurements

The electrochemical measurements were performed with a Metrohm Autolab potentiostat/galvanostat, PGSTAT204 (Metrohm Autolab B.V., Utrecht, The Netherlands), controlled by NOVA 2.1 software. The testing conditions were as follows: Starting potential 0.6 V, switching potential −0.53 V, scanning rate 0.1 V/s, and frequency 100 Hz.

The electrode is immersed in undiluted wine while a potential is applied. This allows the deposition on the electrode surface of the loosely bound fraction of Cu from the wine to form a copper–protein complex. After the electrode is typically immersed in wine for 1, 90, and 180 s, the electrode is washed with 60% aqueous ethanol and then 4.0 M ammonium acetate. The potential of the electrode is then allowed to increase and, at a potential characteristic of copper ions, the resulting cyclic voltammogram is obtained.

For the cyclic voltammetric analysis of the copper concentration in wine it was not necessary to prepare samples, but it was only used at a temperature of 14 °C.

### 2.4. Atomic Adsorption Spectrometry

Spectrophotometric analyses were recorded using the atomic absorption spectrometer (AAS), Schimadzu AA 6300, according to “Compendium of International Methods of Analysis”. The sample preparation procedure for copper ion determination was simple and involved dilution: A 20 mL wine sample was placed in a 100 mL volumetric flask and the volume was made up to 100 mL with double-distilled water. The dilution was modified for some samples to obtain a response within the dynamic range of the detector. The absorbance was measured at 324.8 nm [26].

### 2.5. Scanning Electron Microscopic Analysis

The microscopic analysis of the working electrode before immobilization, after immobilization, and after use in the copper solution was performed with a VEGA II LMU scanning electron microscope (TESCAN, Czech Republic).

### 2.6. Immobilization of Biological Element on SPE and Development of Biosensor

An immobilization of the biological element with benzophenone was carried out in the presence of ultraviolet light; the element was fixed to the working electrode at a wavelength of 350 nm. Then, 1 µL of standard protein solution (200 mg BSA/mL) and 1 µL benzophenone solution 5% were pipetted on the surface of the working electrode of the SPE. The immobilization occurred together (BSA and Ph_2_CO) under the influence of ultraviolet radiation for 20 min, then the miniaturized electrode was introduced into a cell with 10 mL of sample and analyzed by cyclic voltammetry.

### 2.7. Statistical Analysis

Descriptive statistics (standard deviation, coefficient of variation) and the graphs were performed with Origin 8E (OriginLab Corporation, Northampton, MA, USA) software.

## 3. Results

The developed biosensor was based on the following processes: The use of BSA as a biologically active element for the selection of copper ions, photochemical immobilization with benzophenone by irradiation under UV light, and analysis of copper content using these modified screen-printed electrodes with a potentiostat that provides a response in the form of a cyclic voltammogram.

The quantitative analysis of the interaction between copper (II) complexes and bovine serum albumin was undertaken by cyclic voltammetry. These interactions between BSA and copper represent a whole biological phenomenon related to the formation of covalent bonds. Studies of the behavior of BSA have shown a greater affinity for the first copper (II) ion than for those subsequently bound. The binding site for this first copper ion seems to include the amino-terminal position as Peters and Blumenstock have specified [27].

**Figure 1:**
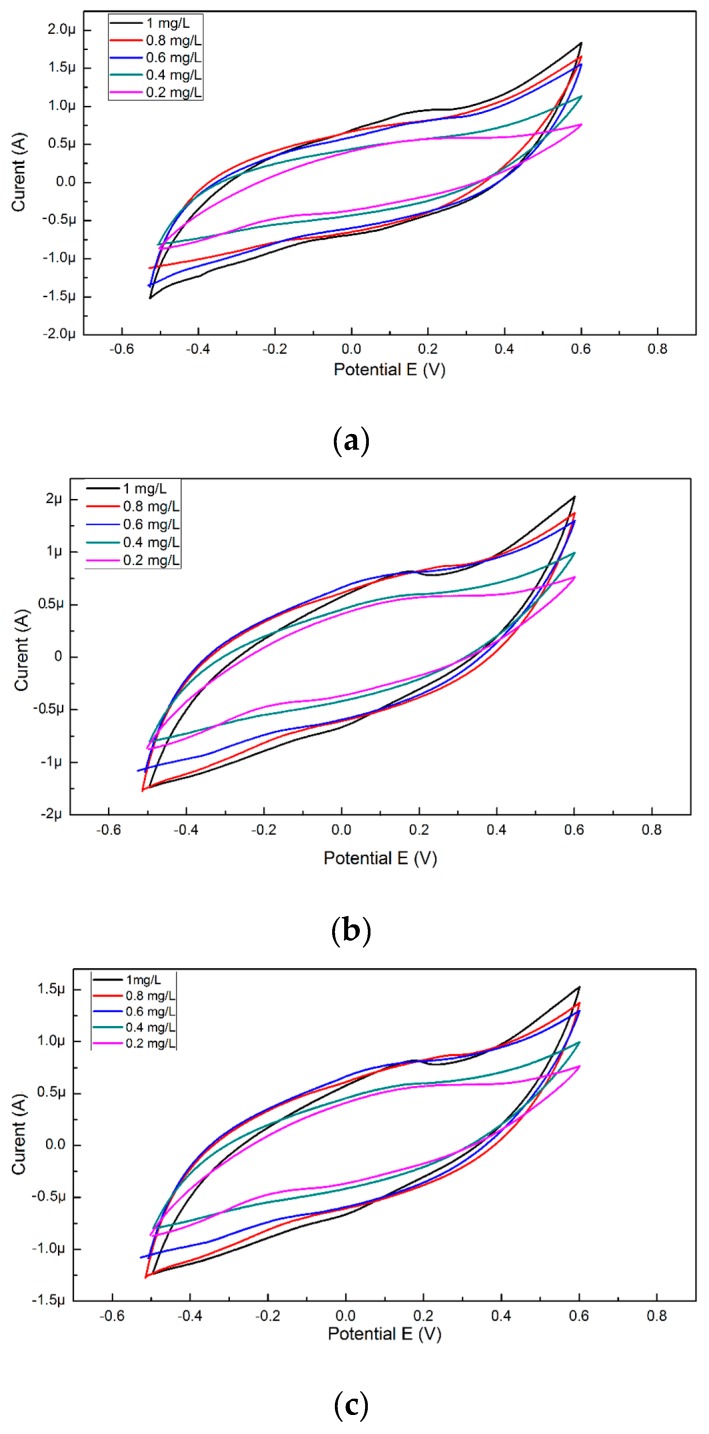
The cyclic voltammogram obtained for the five copper solutions, (**a**) analyzed voltammetrically immediately after the introduction of the electrode into the cell, (**b**) after 90 s from the introduction of the electrode into the cell, (**c**) after 180 s from the introduction of the electrode into the cell.

In order to obtain a calibration curve from the cyclic voltammograms, copper solutions (0.2, 0.4, 0.6, 0.8, and 1 mg/L) were used. The concentration was reported at the maximum current density for each solution because the anodic and cathodic peaks are extremely small. For recording possible changes of the maximum current occurring with time, for the same concentrations of copper, cyclic voltammograms were also performed but analyzed after 90 and 180 s from the time of insertion of the SPE electrode into the cell with solution (Figure 1).

**Figure 2:**
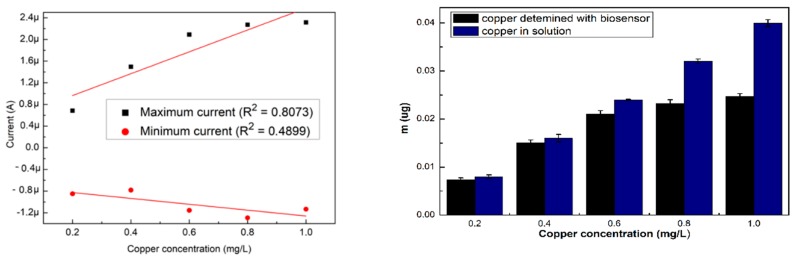

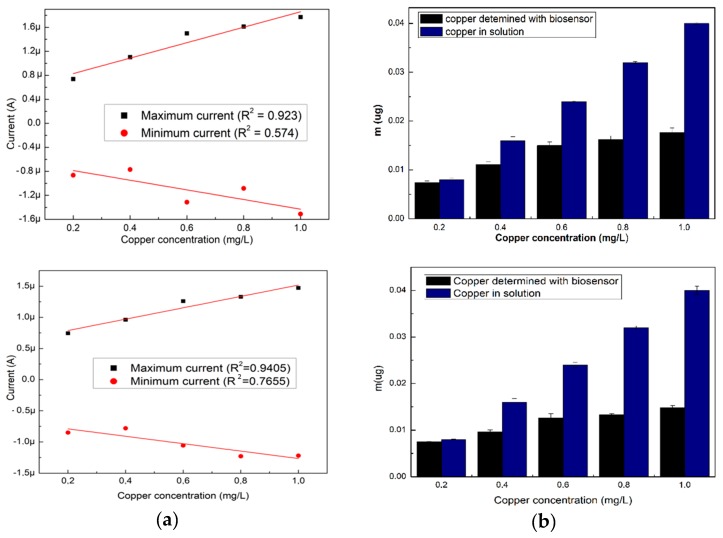
(**a**) Variation of the maximum and minimum intensity of the three voltammograms, and (**b**) amount of labile copper deposited on the SPE, depending on the existing copper concentration. All the analyses were carried out in triplicate.

The NOVA 2.1 software was used for the measurement of the current obtained from the resulted voltammograms, the maximum and minimum intensity values were extracted and used to perform the calibration curves that are shown in Figure 2a. For the specific cyclovoltagrams (with the reduction potential of copper and the Cu–BSA complex), necessary to characterize the performances of the sensor, the option of the triangular signal was selected from the software so that the interpretation of the voltammograms could be realized at the maximum reduction potential (0.6 V). This potential for copper reduction is known from the electrochemical literature and is also on the scale of Nernst potentials. After 180 s, cyclovoltagrams the highest R^2^ (0.94) was observed, while the lowest value of R^2^ (0.80) resulted for cyclic voltammograms performed immediately after the introduction of the electrode into the cell. The highest value of R^2^ at 180 s may be the result of a stability increase of the solution in time. Furthermore, depending on the amount of copper present in the solution (10 mL), the mass deposited on the electrode during the electrochemical measurements was calculated according to Faraday’s law, and the resulting quantities are presented in Figure 2b.

The highest quantities of copper detected with the biosensor were for the electrodes analyzed immediately after the introduction of the electrode into the cell, showing that the evolution in time was not favorable in the sense that the quantities of deposed copper on the SPE were reduced.

**Figure 3:**
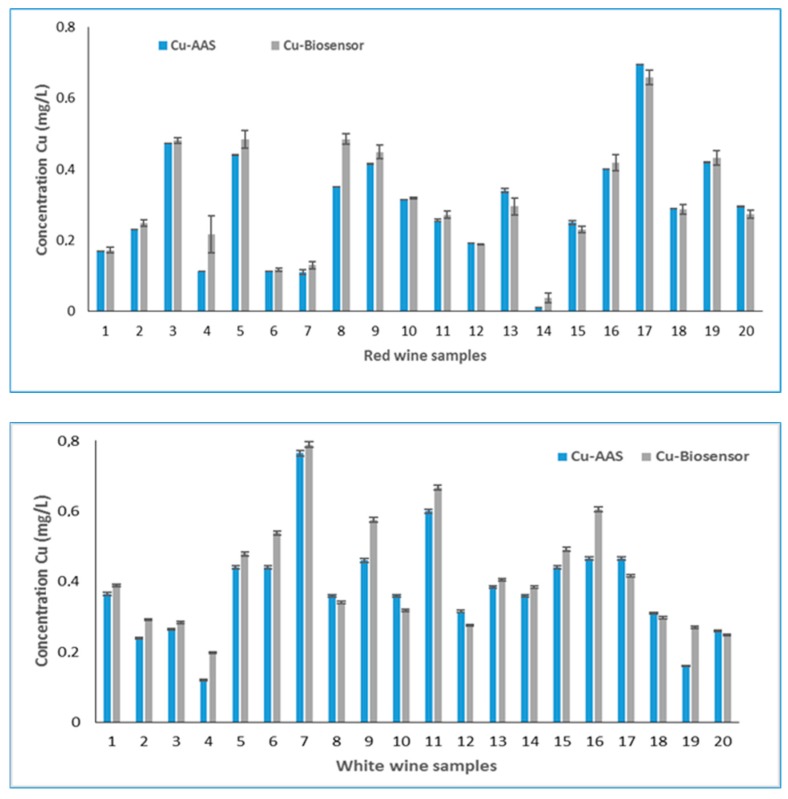
Concentrations of labile copper in red and white wine.

The results obtained for the 20 samples of red wine by voltammetric method and the AAS method are presented in Figure 3.

It can be observed that the differences between the two methods were small enough to recommend the use of the voltammetric method in wine analysis. The effect of pH on the voltammetric response of Cu (II) is well known by the fact that at a lower pH, both the cathodic and anodic peak current are high. Therefore, with the increase of pH, the cathodic and the anodic peak current gradually decrease [28]. This is probably the cause of the higher selectivity of the new method developed, in the case of red wines that have a lower pH (3.29–3.38) than the white wines analyzed (3.59–3.76). The differences between the two methods are more visible for white wine than for red wine, which confirms this assumption.

Another remark was that from the results of 20 samples of white wine analyzed with the developed biosensor, 14 samples had higher values than the AAS method, suggesting that this biologic element may select from the matrix of wine compounds another ion that interferes with copper. Similar, in the case of red wines, of the 20 samples analyzed with the biosensor, 13 samples indicated a higher copper concentration than the AAS reference method. The mean percentage recovery for the repeated analysis was calculated from difference of results obtained with AAS and biosensor depending on the type of wine, and it was about 98.69% for white wine and 96.30% for red wine. The coefficient of variation calculated for the results of two methods was between 0.6 and 3.7 for the red wine and between 0.9 and 1.72 for white wine.

**Figure 4:**
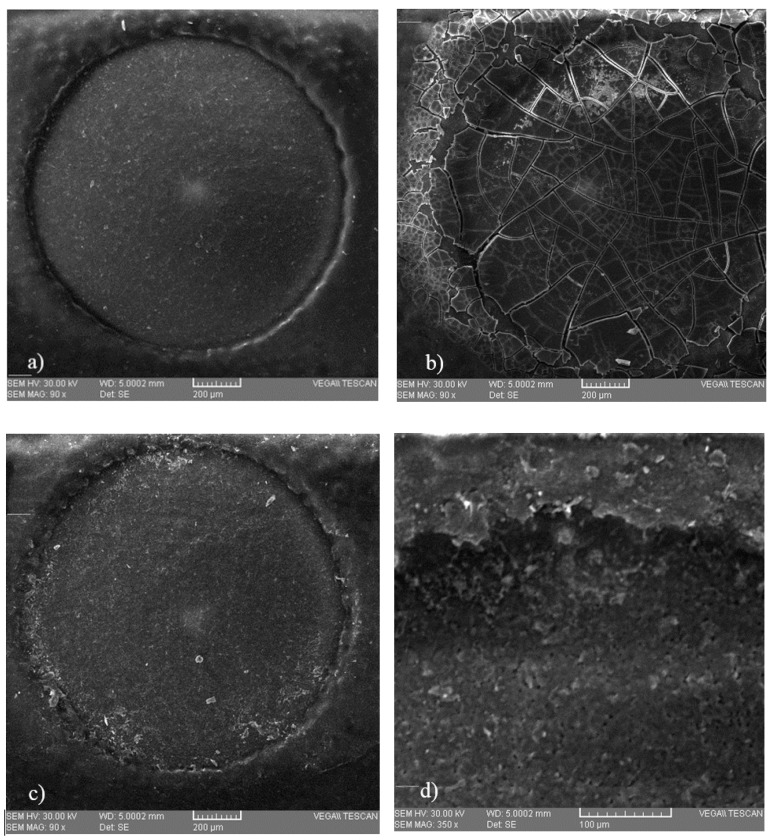
Microscopic images of the surface of the working electrode of (**a**) a screen-printed electrode (SPE) that was not used, (**b**) a SPE containing immobilized bovine serum albumin (BSA), and (**c**) a SPE that was introduced into a 0.6 mg/L copper solution, with a close-up (**d**) obtained at a magnification of 350×.

Microscopic analysis (Figure 4) was performed in order to observe possible deposition on the surface of three SPE electrodes, on an unused electrode, on the surface of an electrode after immobilization of the protein, and on an electrode that was introduced into a copper solution of 0.6 mg/L, respectively. These microscopic images show that some deposits of copper occurred, which means that this method is viable.

### Analytical Performance of the Biosensor

The analytical performances of the biosensor were evaluated by sensitivity, detection limit, and biosensor response time.

Sensitivity was calculated using Equation (1) shown below:

(1) $$Sensitivity=\frac{m}{A},$$

where *m*—slope of calibration curve (μA mM^−1^), *A*—area of active surface (cm^2^) [29].

The detection limit of blank sample (LoB), Equation (2), and the limit of detection (LoD) of the proposed biosensors are terms used to describe the lowest concentration of an analyte that can be reliably measured by an analytical procedure.

*LoB* = *meanblank* + (*SDblank*), (2)

where *SD*—standard deviation.

The LoD (Equation (3)) was determined by using the measured limit of blank sample (LoB) and the standard deviation of the lowest sample concentration [30,31]:

*LoD* = *LoB* + (*SDlow concentration sample*). (3)

The biosensor response time could be influenced by kinetic parameters, diffusion barriers, protein quantity, protein activity, and immobilization procedure [32].

**Figure 5:**
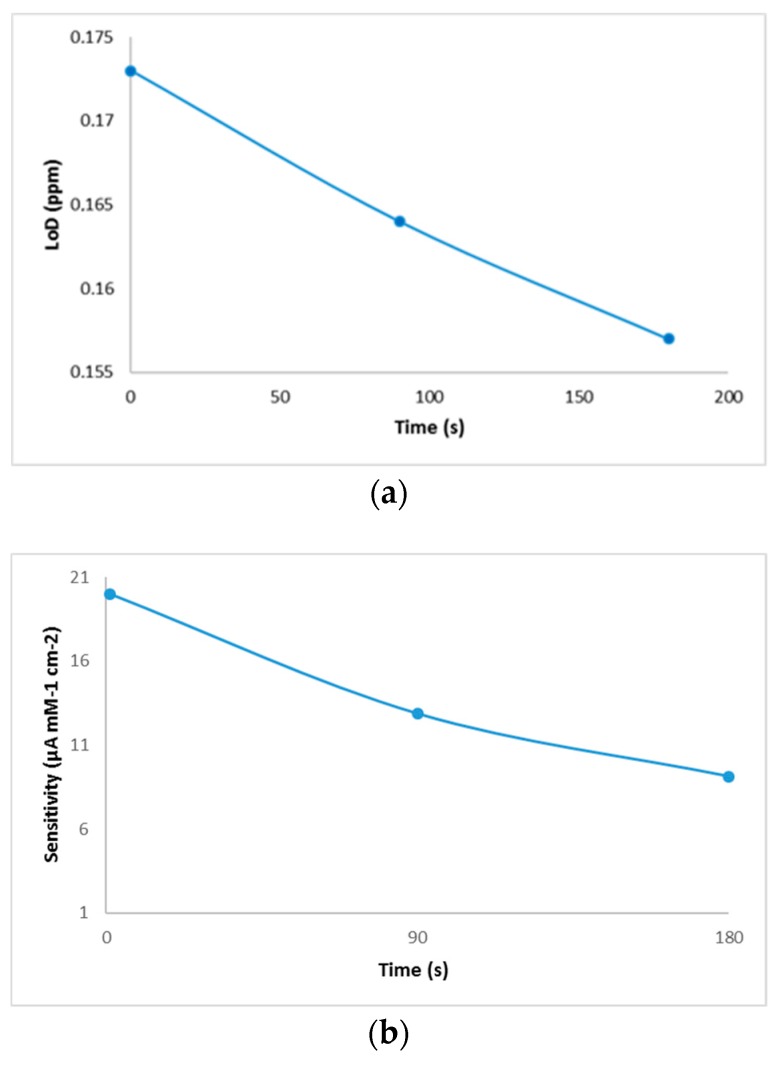

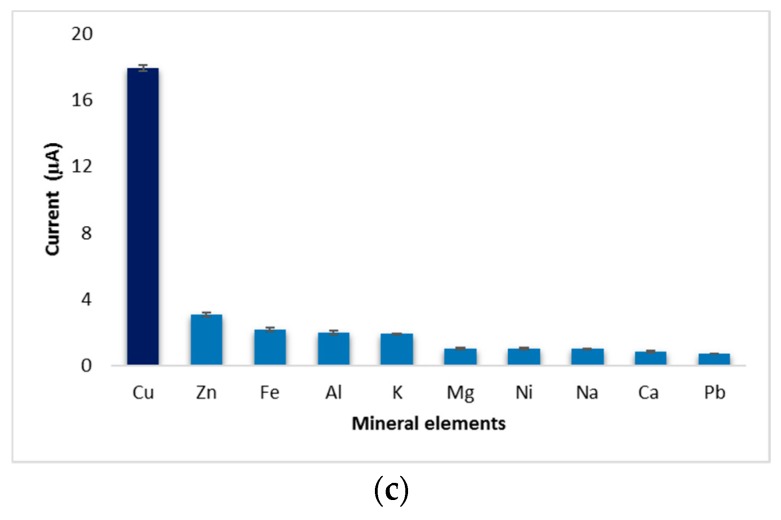
Performance characteristics of the biosensor: (**a**) limit of detection, (**b**) sensitivity, and (**c**) selectivity evaluation of the developed biosensor against the interference of mineral elements present in wine.

Biosensor reproducibility was studied by measuring the copper-generated current of 0.6 mg/L in 10 mL deionized water. The biosensor was tested on three different days with a triple analysis (n = 3). The total mean value was calculated and the relative standard deviation (RSD) provided analytical precision, and, after 90 s from the introduction of the electrode into the cell, the resulted reproducibility was 93.8%. The resulting values of the performance characteristics of the biosensor are shown in Figure 5.

## 4. Discussion

Following the analysis of the copper solutions, higher linear regression values (0.94) were obtained after 180 s of immersion of the electrode and lower values (0.80) for cyclic voltammograms performed immediately after the electrode was inserted into the cell. This phenomenon could explain an increase in the stability of the solution over time. Although, the values of linear regression proved to be better in the case of analyses after 180 s, the calculation of the amount of copper deposited on the electrode proved to be more favorable for those which were analyzed voltammetrically immediately after the introduction of the electrode into the cell. By comparing the developed method with the existing reference method, similar values were observed for both white and red wines. Additionally, this comparison shows that the differences between the two methods were small enough to recommend the use of the voltammetric method in wine analysis. Another evidence that confirms the viability of this new method is provided by the microscopic images that show some copper deposits. Regarding the analytical performance of the biosensor, the highest value of the resulting sensitivity was determined in the case of the sample analyzed when the electrode was introduced into the solution, and it decreased from the time of immersion to the time of the voltammetric analysis. The evolution of the detection limit values with time was the opposite, a fact that brings a major advantage to the performance of the biosensor. The resulting values of LoD were similar to those obtained by other authors [33]. The results of selectivity evaluation of the developed biosensor against the interference of minerals in wine prove that this new method has a good selectivity. Of the minerals analyzed, zinc has a got the highest level detected, which was expected since previous studies indicated its ability of binding to bovine serum albumin [21].

## 5. Conclusions

The results obtained of the proposed biosensor for determining copper ion concentration in wine have proven to be effective both for the performance characteristics and for comparing the results with the reference method. In the experimental part of the study, the biological element used showed good selectivity for the target analyte, and also indicated sensitivity and a detection limit that could recommend it as a possible means for the quantification of copper ions in wine. Therefore, the developed biosensor solves the real problem in quantifying copper ions, having in view that the present methods are laborious, expensive, and cannot be used in in situ analyses.
